# Intelligent metasurfaces: digitalized, programmable, and intelligent platforms

**DOI:** 10.1038/s41377-022-00876-8

**Published:** 2022-08-01

**Authors:** Shuang Zhang

**Affiliations:** 1 Department of Physics, University of Hong Kong, Hong Kong, 999077 China; 2 Department of Electrical and Electronic Engineering, University of Hong Kong, Hong Kong, 999077 China

**Keywords:** Metamaterials, Micro-optics

## Abstract

Distinguished from conventional ones, intelligent metasurfaces are endowed with three important characteristics: digitalization, programmability, and intelligence, which can be further integrated with detection, artificial neural networks, and feedback systems into a smart platform. Metasurface-based smart systems are expected to play an important role in wireless communications, advanced sensing technologies and artificial intelligence.

There has been a recent thrust towards realization of dynamically programmable metasurfaces with independently reconfigurable unit cells^1–10^. This would enable real-time control of the scattering properties of the metasurface. While it still remains a challenge to implement such dynamically tunable metasurfaces at optical frequencies, exciting progress has been made in microwave metasurfaces^1,3,6^, where active electronic elements with large tunability can be incorporated into the metasurface design to achieve dynamic modulation in real time. These metasurfaces can be further integrated with detection, artificial neural networks, and feedback systems into a smart platform to manipulate the wave-information-matter controls without human supervision or intervention^11,12^.

In the review article by Li et al.^13^, the authors provide a thorough and in-depth review on the recent progress of this emerging topic, covering the historical account, basic concept and principles of dynamically tunable metasurfaces. They identified three important characteristics that distinguish intelligent metasurfaces from the conventional ones, namely, digitalization, programmability, and intelligence. Briefly, digitalization refers to information storage in the physical level, reprogrammability means dynamic tunability of individual building blocks, and intelligence requires algorithms for decision making and automated operation. The rapid development in artificial intelligence (AI) provides the theoretical foundation for the intelligent metasurfaces.

The article^13^ discussed the design strategy of the intelligent metasurfaces and identified the coding metasurfaces with a limited number of controllable states (such as binary states) as the most promising approach, considering the benefit of design process, fabrication complexity, cost, and energy consumption. Each subwavelength pixel incorporates an electrically tunable diode, which can toggle between two contrasting states with different reflection phases, leading to a digitalized 2D profile that can be programmed in real time. This digitalized programmable metasurfaces are capable of arbitrarily controlling the wavefront of reflected beam, and they greatly expand the functionalities useful for applications such as beaming, focusing or holographic animations. The active metasurfaces of binary type can convert an input signals of a sequence of 1 and 0 into a certain distribution of the binary states of each individual antennas across the metasurface, leading to powerful manipulation of the reflective beams.

The paper^13^ discusses the progress from dynamically metasurfaces where the task of beam manipulation is preset, i.e. the control digital sequences are calculated and transferred into the FPGA beforehand, to adaptive construction of control sequences in response to the change of ambient environment without human supervision. Two specific examples are given—a smart Doppler cloak with a time-modulated intelligent metasurface integrated with a velocity detector, an arbitrary waveform generator. The progress indicates the immense potential of smart metasurfaces for various applications. Besides beam manipulations, the authors also discussed other potential applications with dynamic metasurfaces, including wireless power transfer, dynamic holograms, and invisibility cloaks.

Intelligent metasurfaces have the great potential to play an important role in wireless communications (see Fig. 1 for its potential applications), as the information capacity could be improved compared to conventional approaches. The authors envisioned two scenarios of metasurface-aided wireless communication: recycling and redirecting of RF signal towards the users, and encoding of additional information to the intelligent metasurfaces. They further categorized the communication architectures into three major types: (A) non-modulated-metasurface backscatter communications, (B) modulated-metasurface backscatter communications, and (C) ambient modulated-metasurface backscatter communications. Detailed discussions on how these three different types of systems operate are provided, along with the advantages and disadvantages of each type.

**Fig. 1:**
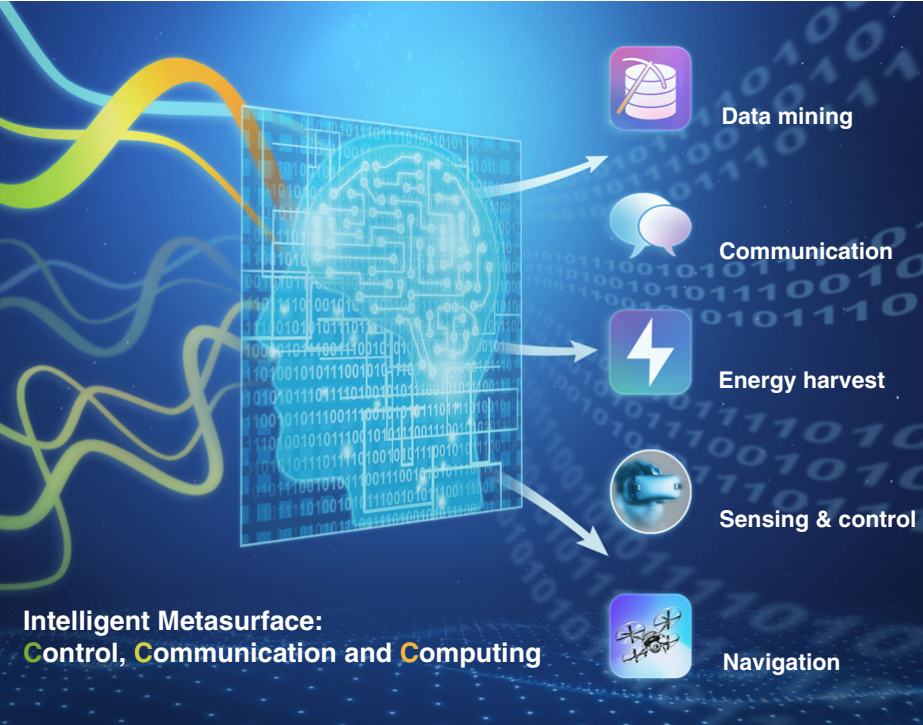
Future applications for intelligent metasurfaces.

The article further discussed the applications of intelligent metasurfaces on computing and sensing. There have been recent efforts to use wave-based analog systems to perform computing. Their main advantages compared to the conventional electronic digital computing include parallelism and low energy consumption. Intelligent metasurfaces provide a new platform for robot human alliance, i.e., design of robots that can understand human behaviors and build a digital recognition map of the physical world through sensing and information processing. These include remote sensing of human locations, activities, physiological states, etc. The authors identified three different schemes of intelligent sensing involving intelligent metasurfaces, including digital-computing-free intelligent sensing, hybrid-computing-based sensing and hybrid-computing-based integrated sensing.

Finally, the authors provide an outlook on future directions of this field, such as more specialized intelligent metasurfaces for lower power consumption, integration between artificial intelligence and active artificial materials, and the ultimate goal of all-wave information systems.

This is a very timely review that covers a very promising research area. It not only presents a comprehensive review of the recent progress of intelligent metasurfaces to which the authors have made significant contributions but also provides some new insight into how the field may evolve in the future. With the fast development in material science and nanofabrication techniques, intelligent metasurfaces may also be extended to higher frequencies (e.g., terahertz, infrared, and optical) so the information capacity will be further enhanced with a more compact platform.

## References

[1] Cui T (2014). Coding metamaterials, digital metamaterials and programmable metamaterials. Light Sci. Appl..

[2] Li L (2017). Electromagnetic reprogrammable coding-metasurface holograms. Nat. Commun..

[3] Yu Yao Raji (2014). Electrically tunable metasurface perfect absorbers for ultrathin mid-infrared optical modulators. Nano Lett..

[4] Zhang L (2018). Space-time-coding digital metasurfaces. Nat. Commun..

[5] Liu S (2016). Anisotropic coding metamaterials and their powerful manipulation of differently polarized terahertz waves. Light Sci. Appl..

[6] Cui TJ, Liu S, Li LL (2016). Information entropy of coding metasurface. Light Sci. Appl..

[7] Chen K (2017). A reconfigurable active Huygens' metalens. Adv. Mater..

[8] Nemati A, Wang Q, Hong MH, Teng JH (2018). Tunable and reconfigurable metasurfaces and metadevices. Opto-Electron Adv.

[9] Yang H (2016). A programmable metasurface with dynamic polarization, scattering and focusing control. Sci. Rep.

[10] Gao LH (2015). Broadband diffusion of terahertz waves by multi-bit coding metasurfaces. Light Sci. Appl..

[11] Ma Q (2019). Smart metasurface with self-adaptively reprogrammable functions. Light Sci. Appl..

[12] Li L (2019). Intelligent metasurface imager and recognizer. Light Sci. Appl..

[13] Li L (2022). Intelligent metasurfaces: control, communication and computing. eLight.

